# Pediatric tizanidine toxicity reversed with naloxone: a case report

**DOI:** 10.1186/s12245-021-00397-y

**Published:** 2021-12-14

**Authors:** Doaa Bader, Ahmed Adam, Mohamed Shaban, Bader Alyahya

**Affiliations:** 1 Royal Commission Medical Center, Yanbu, 46451 Saudi Arabia; 2 Emergency Medicine Department, King Saud University Medical City, King Saud University, Box 2925, Riyadh, PO 11461 Saudi Arabia

**Keywords:** Tizanidine, Naloxone, Toxicology, Pediatric

## Abstract

**Background:**

Tizanidine, an α-2 adrenoceptor agonist, is widely prescribed for the management of spasticity in adults. Case reports on pediatric tizanidine overdose are limited. Here, we report a case of pediatric tizanidine toxicity that was reversed with naloxone.

**Case presentation:**

A 3-year-old male presented to the emergency department with lethargy, bradycardia, and bradypnea after accidental ingestion of multiple tizanidine tablets. Improvements in the level of consciousness and respiratory and heart rates were observed after two intravenous administrations of naloxone at a dose of 0.05 and 0.1 mg/kg, respectively.

**Conclusions:**

This case report provides further evidence regarding the use of naloxone as a viable antidote for centrally acting α-2 receptor agonists and presents additional epidemiologic data on childhood tizanidine poisoning.

## Background

Tizanidine hydrochloride is a centrally acting imidazoline derivative with α-2 adrenoceptor agonist properties. It is widely prescribed for the management of spasticity-related conditions, including multiple sclerosis, cerebral palsy, spinal cord injuries, and regional musculoskeletal pain syndromes [1–3]. Tizanidine activates presynaptic α-2 adrenoceptors within the central nervous system, thereby inhibiting the release of the excitatory neurotransmitter norepinephrine via a negative feedback mechanism [4].

Tizanidine overdose usually results in hypotension and bradycardia [1, 5]. Currently, there is no known antidote for tizanidine intoxication, and treatment options are limited to endotracheal intubation and administration of intravenous fluids and vasopressors as necessary [6, 7].

Central α-2 receptor agonist toxicity has been well documented in numerous case series and case reports [8, 9], with effects ranging from central nervous system depression, bradycardia, hypotension, miosis, and hypothermia. They have also been suggested as a “one pill can kill” drug when adult doses are unintentionally ingested by children [10].

A retrospective investigation of unintentional pediatric exposure to central α-2 receptor agonists indicated a significant increase in tizanidine intoxication over an 11-year period [8]. A variety of treatments including atropine, intravenous fluids, naloxone, and vasopressors, were used in these cases illustrating that one specific therapy was not highly effective.

There is a paucity of case reports on tizanidine overdose or toxicity, especially in children. Here, we describe a case of pediatric tizanidine toxicity that was reversed with naloxone.

## Case presentation

A previously healthy 3-year-old male (14 kg) presented to the emergency department with lethargy, decreased level of consciousness, and difficulty breathing for one hour. He was found near scattered tablets of tizanidine (4 mg each) about an hour prior to presentation and had become pale and drowsy over the ensuing 30 min. His condition warranted emergency care. The exact amount of ingested tablets was unknown, but his parents indicated four to five missing tablets.

Upon examination, the toddler appeared ill and lethargic. His pupils were constricted to 2 mm in diameter, and his level of consciousness was reduced with a Glasgow coma scale (GCS) score of 10/15. His temperature was 36.2 ^o^C, blood pressure was 93/44 mmHg (reference range systolic pressure from 88 to 109 mmHg, diastolic pressure from 45 to 64 according to the blood pressure level by age and height percentile ), heart rate was reduced to 56/min (reference range 80–120/min), respiratory rate was depressed to 10/min (reference range 20–30/min), and oxygen saturation was 99% on room air. His serum glucose concentration was 133 mg/dl. His breathing and heart sounds were normal. There was no murmur. Abdominal examination was normal. Electrocardiography revealed sinus bradycardia. His venous blood gas analysis demonstrated a pH of 7.32, Pco_2_ of 45.5 mmHg, and HCO_3_ of 23.6 mEq/L. Results of renal and liver function tests, and urine analysis were all within the normal ranges.

The patient was admitted to the pediatric intensive care unit where he was administered non-invasive mechanical ventilation (bilevel positive airway pressure [BiPAP] using the following settings: positive end-expiratory pressure 5 cmH_2_O, peak inspiratory pressure 10 cmH_2_O, respiratory rate 20/min, and fraction of inspired oxygen 30%) for approximately 90 min. However, the patient became increasingly lethargic and miosis worsened to “pin-point pupils.” The respiratory rate reduced to 7 breaths per minute, which prompted a trial of naloxone at a dose of 0.05 mg/kg intravenously. This resulted in spontaneous regaining of consciousness 60 s post-administration after which the BiPAP mask was removed. His heart and respiratory rates increased to 90/min and 16/min, respectively.

However, 40 min after the initial administration of naloxone, both heart and respiratory rates decreased to 60/min and 10/min, respectively. A second dose of naloxone at 0.1 mg/kg was administered intravenously and the child became fully alert (GCS score of 15) 60 s after administration. Both heart and respiratory rates increased to 90/min and 12/min, respectively. The diameter of the pupils increased to 4 mm and both pupils were equally reactive.

The patient remained in the pediatric intensive care unit for 6 h and was then transferred to the pediatric ward for observation for a further 12 h. He was discharged without complications. His blood pressure and heart rate at the time of discharge were 90/64 mmHg and 125/min, respectively. There were no subsequent hospital visits related to this event 4 months after discharge.

## Discussion

Acute intoxication with central α-2 receptor agonists is a recognizable clinical entity with signs and symptoms that largely resemble opiate intoxication, prompting the use of naloxone hydrochloride as a reversal agent for this type of poisoning. The therapeutic mechanism of naloxone is thought to involve competing with endogenous opioids, leading to an increase in sympathetic tone [6].

There have been a few case reports over the past decades in which naloxone has been described as a viable agent for acute α-2 receptor agonist toxicity. For example, one report stated that a single 0.1 mg dose of naloxone was able to reverse tetrahydrozoline toxicity in a 36-month-old toddler within 30 s of administration [11]. Another similar case report described resolution of symptoms of tetrahydrozoline toxicity after one-time administration of 0.1 mg of naloxone in a 25-day-old infant [12].

Successful treatment with naloxone has also been documented in unintentional pediatric clonidine and guanfacine exposures [13–15]. However, it should be noted that the use of naloxone remains controversial, with multiple reports stating its inefficacy [4, 16].

Even though use of naloxone for pediatric clonidine, brimonidine, guanfacine, and tetrahydrozoline poisoning has been widely reported, only one retrospective study reports its use in pediatric tizanidine poisoning [8]. However, the authors of this study did not comment on the efficacy of naloxone in pediatric tizanidine poisoning. Moreover, literature review revealed only one other case of definitive response to naloxone in tizanidine overdose; however, the authors did not specify the age of this patient^5^. Therefore, to the best of our knowledge, this is the first case report that documents successful treatment of tizanidine toxicity using naloxone in a pediatric patient. To this end, this case report provides further evidence regarding the use of naloxone as a viable antidote for centrally acting α-2 receptor agonists and presents additional epidemiologic data on childhood tizanidine poisoning.

## Data Availability

The data is available in the patient’s medical record.

## Abbreviations

GCS: Glasgow coma scale
BiPAP: Bilevel positive airway pressure
